# Uncovering Knowledge Gaps in the Safety Profile of Antiangiogenic Drugs in Cancer Patients: Insights from Spontaneous Reporting Systems Studies

**DOI:** 10.3390/ph16060867

**Published:** 2023-06-12

**Authors:** Valerio Ciccone, Marina Ziche, Andrea Spini, Sandra Donnini

**Affiliations:** 1 Department of Life Sciences, University of Siena, Via Aldo Moro 2, 53100 Siena, Italy; valerio.ciccone@unisi.it; 2 Department of Medicine Surgery and Neuroscience, University of Siena, Viale Mario Bracci 16, 53100 Siena, Italy; marina.ziche@unisi.it; 3 Azienda Ospedaliera Universitaria Senese, Viale Mario Bracci 16, 53100 Siena, Italy

**Keywords:** angiogenesis, adverse drug reaction, VEGF, disproportionality analysis, postmarketing surveillance, spontaneous reporting systems

## Abstract

Global repositories of postmarketing safety reports improve understanding of real-life drug toxicities, often not observed in clinical trials. The aim of this scoping review was to map the evidence from spontaneous reporting systems studies (SRSs) of antiangiogenic drugs (AADs) in cancer patients and highlight if the found disproportionality signals of adverse events (AEs) were validated and thus mentioned in the respective Summary of product Characteristics (SmPC). This scoping review was conducted according to PRISMA guidelines for scoping reviews. A knowledge gap on the safety of AADs was found: firstly, several cardiovascular AEs were not mentioned in the SmPCs and no pharmacovigilance studies were conducted despite the well-known safety concerns about these drugs on the cardiovascular system. Second, a disproportionality signal (not validated through causality assessment) of pericardial disease was found in the literature for axitinib with no mention in SmPC of the drug. Despite the exclusion of pharmacoepidemiological studies, we believe that this scoping review, which focuses on an entire class of drugs, could be considered as a novel approach to highlight possible safety concerns of drugs and as a guide for the conduction of a target postmarketing surveillance on AADs.

## 1. Introduction

Tumor angiogenesis plays a significant role in the development and progression of cancer. Tumors require a blood supply to grow and spread, and they often secrete proteins that stimulate angiogenesis to create new blood vessels. As a result, targeting angiogenesis has become an important therapeutic approach in oncology. A key proangiogenic mediator is vascular endothelial growth factor (VEGF), but other factors can stimulate angiogenesis including fibroblast growth factor-2 (FGF-2), platelet derived growth factor (PDGF), hepatocyte growth factor (HGF), angiopoietins and inflammatory mediators, such as interleukins and prostaglandins [1]. Antiangiogenic drugs are a class of drugs designed to prevent the formation of new blood vessels or to inhibit the growth of existing ones. Antiangiogenic drugs (AADs) include VEGF/VEGFR-directed monoclonal antibodies, small molecules kinase inhibitors and the VEGF-Trap aflibercept. The two categories of AADs present different pharmacological profiles: monoclonal antibodies and derivatives, which are injected and target the extracellular part of VEGFR or the soluble VEGF, and protein kinase inhibitors (PKIs), which are orally taken small molecules targeting the intracellular ATP domain of different kinases. Indeed, PKIs can regulate a single pathway involved in angiogenesis, such as axitinib for VEGFR-1/2/3, or multiple signaling pathways including tyrosine and serine/threonine kinases (ponatinib, regorafenib and sorafenib) (Table 1) [2]. The multi-inhibition strategy was developed to avoid resistance to anti-VEGF drugs and improve efficacy [3]. They have become an important part of some tumors’ treatment, both in monotherapy or in combination [4,5,6].

By inhibiting the formation of new blood vessels, antiangiogenic drugs deprive tumors of the nutrients and oxygen they need to grow and spread [7]. In oncology, antiangiogenic drugs have been used in the treatment of various types of cancer, including lung, breast, colon, kidney and brain cancers. In several advanced tumors, they are often used in combination with chemotherapy or radiation therapy, targeted therapies and immune therapies to enhance their effectiveness [5]. However, inhibition of VEGF signalling and the angiogenesis process involves a number of adverse events, and AADs, therefore, have a number of common adverse events, such as hypertension, heart failure, thromboembolic events, bleeding and impaired wound healing, the severity of which can significantly reduce the quality of life and life expectancy of special populations (e.g., pediatric patients, the elderly, women). The use of AADs in special populations requires careful consideration of the individual patient’s characteristics and the potential risks and benefits of treatment. Close monitoring and management of side effects may be necessary to ensure safe and effective treatment [6]. Despite the challenges, AADs have shown promise in the treatment of cancer and continue to be an active area of research [8,9,10].

Postmarketing safety surveillance of drugs is essential, as not all adverse effects (AEs) of a drug may be identified from premarketing clinical trials. The spontaneous reporting systems (SRSs) play an important role in pharmacovigilance by providing information from real-life clinical setting throughout the life of a drug. SRSs are a widely used, effective, and relatively inexpensive method of collecting information on suspected adverse drug reactions (ADRs). Their main function is the detection of new, rare, and serious ADRs which remained undetected in the premarketing clinical trials. The systems provide information from real-life clinical practice as opposed to clinical trials, where vulnerable individuals are excluded and the duration of treatment is limited [11]. Evidence coming from clinical trials and pharmacoepidemiology studies on the safety of angiogenesis inhibitors shows that the most common side effects of treatment are hemorrhage, hypertension, impaired wound healing, leukoencephalopathy syndrome, protein in the urine and hematological disorders [12,13]. However, as AADs have shown promise in the treatment of cancer and its use has been growing in recent years as chemosensitizers in combination with chemotherapy/target-therapies/immunotherapies, an overview of the safety signals published in the literature and coming from the SRSs is needed. The objective of this scoping review is to map all the evidence from studies using SRSs studying safety of AADs in patients with cancer and highlight if eventual safety signals from SRS studies have been included in the respective Summary of Product Characteristics (SmPCs).

## 2. Results

### 2.1. Study Characteristics

**Figure 1:**
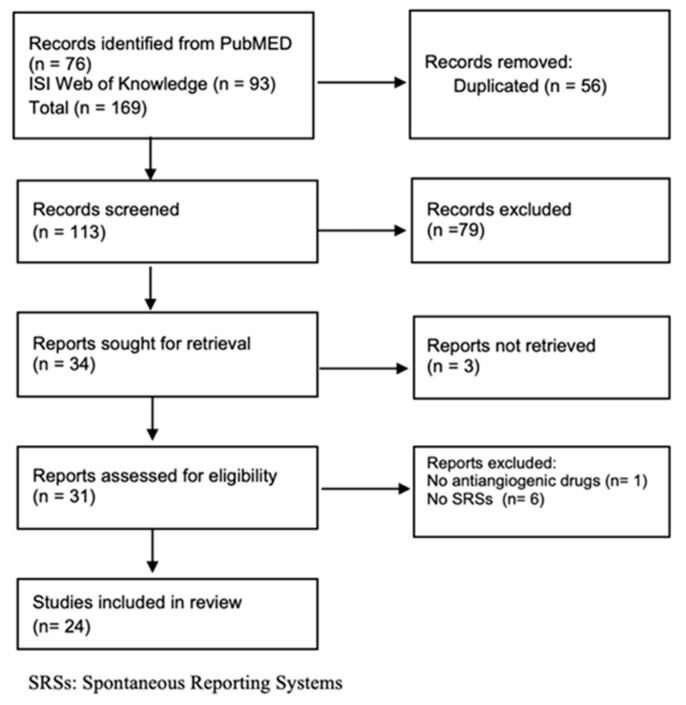
Flow chart of SRS studies.

In total, 76 and 93 articles were retrieved from PubMed and ISI Web of Knowledge, respectively. After duplicate removal and title/abstract screening, 31 available full texts were evaluated for inclusion. In total, 24 articles were included in this scoping review (six were excluded because they did not use SRSs and one because it did not report information on AADs) (Figure 1).

Half of the studies (12 out of 24) used spontaneous reports from the FDA adverse events reporting system (FAERS) database [14,15,16,17,18,19,20,21,22,23,24,25], six from Vigibase [26,27,28,29,30,31], three from the French pharmacovigilance database [32,33,34], two from the Japanese adverse drug report (JADERS) database [35,36] and one from the Italian spontaneous reporting system [37]. Bevacizumab was the most studied drug (13 studies) followed by sunitinib (11 studies), sorafenib (10 studies) and axitinib (nine studies). Two studies also reported the safety of AADs in combination with immune checkpoint inhibitors [14,19]. Most studies performed disproportionality or logistic regression analysis (17 out of 24). See Table 2 for study characteristics.

### 2.2. Cardiovascular Adverse Events in SRS Studies

As these drugs impact vascular homeostasis, 16 studies focused on cardiovascular adverse events (CVAEs). Twelve out of the 16 studies performed disproportionality analysis or logistic regression, while three were descriptive studies. Egron et al. conducted the only study that evaluated the preventability of CVAEs. Disproportionality values are collected in Figure S1. To gain insight into evaluation of cardiovascular safety profiles of AADs belonging to different categories, a stratification between biological and small molecules has been performed. Bevacizumab, ramucirumab and aflibercept were included in the biological group. Five studies focused on cardiovascular AEs of biological drugs [14,22,23,34,37], while seven studied the safety of small molecules [16,17,19,24,26,32,33]. Four studies assessed CVAEs of AADs irrespective of their chemical structure [15,20,27,28].

The study by Wittayanukorn et al. analyzed the FAERS database for any CVAEs with targeted therapies and their combinations versus all other drugs. The authors found that bevacizumab showed the highest Reporting Odds Ratio (ROR) in monotherapy (ROR  =  3.17, 95% CI  =  2.70–3.71) as well as in combination with doxorubicin (ROR  =  8.76, 95% CI  =  6.30–12.18) and cyclophosphamide (ROR  =  6.71, 95% CI  =  4.95–9.10) [22].

Cardiac disorders: Taugourdeau-Raymond et al. performed a descriptive analysis of bevacizumab-associated serious AEs recorded in the French Pharmacovigilance database. The authors found that most cardiological events were at least grade three (6.9%) and were associated with heart failure and arrhythmia [34]. Bevacizumab was also overreported for heart failure by Cutroneo et al. (PRR = 4.69, 95% IC = 3.24–6.79).

Concerning cardiac arrhythmia, Ye et al. analyzing the safety of PKIs, reported a nearly 3-fold increase in the ROR of atrial fibrillation for ponatinib (ROR = 3.03, 95% CI = 2.21–4.14) [24], while a prolongation of the QT interval was reported by Goldman only for lenvatinib (ROR = 2.15, 95% CI = 1.44–3.21) [17].

In the case of heart failure, Goldman et al. found an overreporting for lenvatinib (ROR = 2.21, 95% CI = 1.5–3.27) and sunitinib (ROR = 2.01, 95% CI = 1.68–2.4), and de Campaigno et al. found a ROR for sunitinib of 1.67 (95% CI = 1.51–1.84). Cirmi et al. found a disproportionality signal of ponatinib for heart failure (ROR = 1.8, 95% IC = 1.4–2.4).

As for ischemic heart disease, Goldman et al. reported a disproportionality signal for lenvatinib (ROR = 1.41, 95% CI = 1.19–1.67), Cirmi et al. for ponatinib (ROR = 2.9, 95% CI = 2.4–3.5) and Cutroneo et al. for bevacizumab (PRR = 5.63, 95% CI = 3.98–7.96). No disproportionality signals were found for all other drugs or for bevacizumab by Governeur et al.

Pericardial diseases were overreported with sunitinib and axitinib treatment [ROR = 3.15 (95% CI 2.54–3.91), ROR = 2.15 (1.33–3.46)], respectively [17].

Bai et al. found an OR of 0.63 (95% CI 0.20–1.98) for pericardial effusion when comparing anti-PD-L1 combined versus bevacizumab with anti-PD-L1 monotherapy [14].

Finally, the immune-checkpoint inhibitors (ICIs)–axitinib combination showed a significant increase in myocarditis reporting compared to ICIs alone [(pembrolizumab alone ROR = 24.1, 95% CI = 19.7–29.4, avelumab alone: ROR = 16.6, 95% CI = 4.1, 66.8); pembrolizumab + axitinib (ROR = 36.9, 95% CI = 11.8–115.9); and avelumab + axitinib (ROR = 55.6, 95% CI = 13.4–222.3]). To compare avelumab and pembrolizumab monotherapy to axitinib monotherapy, the FAERS/AERS database was searched for axitinib monotherapy terms. Interestingly, only one report of myocarditis in 5492 axitinib monotherapy reports was found [19].

Aortic aneurism or dissection: Cheng et al. performed a case series study including a total of 240 cases of arterial aneurysm/dissection events reported during treatment with a VEGF inhibitor. They revealed that an arterial aneurysm/dissection event was cited as the cause of death in 22% (n = 53) of cases, including ten cases related to autopsy findings [15]. The median time to onset of an aneurysm/arterial dissection event from the start of na AADs administration was 94 days (range 1–1955 days). For bevacizumab, the authors reported a higher reporting rate among females, while for all the other drugs the trend was the opposite, with a higher reporting rate among males.

Goldman et al. found a disproportionality signal of aortic dissections following treatment with lenvatinib [ROR = 7.14 (4.22–12.08)], sunitinib [ROR = 6.16 (4.37–8.70)] and axitinib [ROR = 4.47 (2.13–9.40)] (Figure S1). Similarly, Wang found an overreporting of aneurysm and arterial dissection for sorafenib (ROR = 1.41, 95% CI = 1.02–1.93), sunitinib (ROR = 1.93, 95% CI = 1.59–2.36), ponatinib (ROR = 2.03, 95% CI = 1.01–4.06), nintedanib (ROR = 2.12, 95% CI = 1.43–3.14), lenvatinib (ROR = 3, 95% CI = 2.11–4.28), bevacizumab (ROR = 3.05, 95% CI = 2.67–3.48) and ramucirumab (ROR = 3.68, 95% CI = 2.18–6.23) [17]. In agreement with these two studies, signals of disproportionality reporting of artery dissections or aneurysms were also found by Guyon et al. for all AADs when compared with all other anticancer drugs (both biological and small molecules) used as the reference group in VigiBase database. When analyzing by drug class, no differences were found [27]. Lenvatinib showed the highest reporting odds ratios among all PKI drugs (PRR = 4.17, 95% CI, 2.30–7.56), followed by axitinib (PRR = 2.52, 95% CI, 1.51–4.19) and sunitinib (PRR = 2.38, 95% CI, 1.87–3.01). Of note, no disproportionality signal was observed for sorafenib, which also inhibits serine/threonine kinases. As for the biological agents, for bevacizumab, a disproportionality signal was found for both arterial [PRR = 4.08, 95% CI, 3.54–4.70] and aortic aneurysm or dissection, while for ramucirumab, only a disproportionality signal was reported for aortic dissection (PRR = 3.34, 95% CI, 1.89–5.90). Aflibercept showed no reporting.

Hypertension: Regarding vascular disorders, hypertension is consistently reported as the most frequent CVAE, with an incidence of 20 and 70% of patients. Cirmi et al. found a disproportionality signal for ponatinib (ROR = 3.5, 95% CI = 2.9–4.3), Goldman et al. with all drugs analyzed [lenvatinib (ROR = 16.74, 95% CI = 15.7–17.7), axitinib (ROR = 6.5, 95% CI = 5.93–7.13), sunitinib (ROR = 5.75, 95% CI = 6.06–6.45), pazopanib (ROR = 4.49, 95% CI = 4.23–4.77) and cabozantinib (ROR = 4.21, 95% CI = 3.91–4.53)]. Yagi et al. also detected a disproportionality signal for bevacizumab (ROR = 2.45, 95% CI = 2.33–2.59) [23] and Cutroneo et al. found a disproportionality signal for bevacizumab (ROR = 11.14, 95% CI = 10.01–12.39) and aflibercept (ROR = 10.09, 95% CI = 6.39–15.95) [37].

Thromboembolic events: Lenvatinib, cabozantinib and sunitinib showed an overreporting of venous embolic events according to Goldman et al. [ROR = 2.79, 95% CI = 2.36–3.3 for lenvatinib, ROR = 1.78, 95% CI = 1.52–2.07 for cabozantinib and ROR = 1.19, 95% CI = 1.02–1.39 for sunitinib)]. A signal of disproportion for ponatinib on venous embolic events was found by Cirmi et al. (ROR = 1.4, 95% CI = 1.2–1.6), while for bevacizumab and aflibercept by Cutroneo et al. [(PRR = 15.18, 95% CI = 13.78–16.73 and (PRR = 4.38, 95% CI = 2.01–9.57, respectively)].

Cerebral vascular disorders: Regarding cerebral disorders, bevacizumab revealed a significant disproportionality signal of vascular disorders according to Cutroneo et al. (PRR = 2.44, 95% CI = 1.87–3.20) and of cerebral aneurism according to Guyon et al. (PRR = 3.76, 95% CI = 2.92–4.84), followed by axitinib and sunitinib (PRR = 2.52, 95% CI = 1.04–6.08 and PRR = 1.89, 95% CI = 1.19–2.99, respectively). Goldman et al. found an overreporting of cerebral ischemia following treatment with lenvatinib (ROR = 2.19, 95% CI = 1.87–2.55) and axitinib (ROR = 1.53, 95% CI = 1.25–1.88). Accordingly, Guyon found an overreporting for axitinib and sunitinib (PRR = 2.52, 95% CI = 1.04–6.08 and PRR = 1.89, 95% CI = 1.19–2.99, respectively). For lenvatinib, a disproportionality signal was found for brain hemorrhages (ROR = 3.32, 95% CI = 2.74–4.02) [17]. The study by Goldman et al. found that lenvatinib was found with an increased reporting of cerebral ischemia (ROR = 2.19, 95% CI = 1.87–2.55) followed by axitinib treatment (ROR = 1.53, 95% CI = 1.25–1.88). No disproportionality signals were found for all other drugs [17].

Concerning biological drugs, bevacizumab revealed a significant disproportionality signal of vascular disorders according to Cutroneo et al. (PRR = 2.44, 95% CI = 1.87–3.20) and of cerebral aneurism according to Guyon et al. (PRR = 3.76, 95% CI = 2.92–4.84).

In addition, the safety of AADs was also studied by stratifying for the elderly and women. Yagi et al. found that bevacizumab was associated with an increased incidence of hypertension in the FAERS database, regardless of age and sex (ROR = 1.66, 95% CI = 1.51–1.84 for male and ROR = 2.38, 95% CI = 2.21–2.56 for female) [23]. Gouverneur et al. compared AEs for AADs used for metastatic colorectal cancer using the VigiBase database by age (<75 vs. ≥75 years). In both age groups, cardiac disorders such as heart failure or ischemic coronary artery disorders were not associated with bevacizumab in comparison with other anticancer drugs [28]. Stratification by sex revealed that compared to men, women had a higher reporting of any CVAE [4312 (26%) vs. 6836 (22%), *p* < 0.001], particularly hypertension [2288 (14%) vs. 3001 (10%), *p* < 0.001 [28].

### 2.3. Cardiovascular Adverse Events in Summary of Product Characteristics

In each SmPC of AADs, both monoclonal antibodies and derivatives, PKIs and CVAEs were usually mentioned as possible complications.

**Figure 2:**
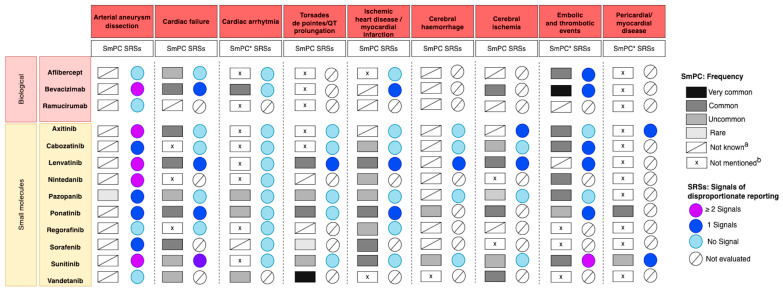
Overview of the frequency of adverse cardiovascular reactions coming from SmPCs and disproportionality signals coming from pharmacovigilance SRS studies of AADs grouped by class. ^a^ The frequency of the event is classified as not known in Section 4.8 “undesirable effects” of the SmPC or it is reported but it is not clear (e.g., (1) if the SmPCs reported haemorrhage without specifying the type, frequency of cerebral haemorrhage was considered as not known, (2) acute myocardial infarction was included in the list of other thromboembolic events). ^b^ Not mentioned in Section 4.8 “undesirable effects” of the SmPCs. * As for cardiac arrhythmia, the following adverse events were the most frequent: tachycardia, atrial fibrillation and bradycardia. As for embolic and thrombotic events, the following adverse events were the most frequent: venous thromboembolism, arterial thromboembolism and thrombosis. As for pericardial/myocardial disease, the following adverse events were the most frequent: pericarditis, myocarditis, pericardial effusion, cardiomyopathy.

Regarding aneurysm or arterial dissection, the frequency was unknown for all drugs evaluated, except pazopanib (rare) [38] (Figure 2).

For heart failure, a common frequency was reported in the SmPC for several AADs, biological agents and PKIs (axitinib [39], lenvatinib [40], ponatinib [41], sorafenib [42] and bevacizumab [43]) while an uncommon frequency was reported for pazonapib [38], sunitinib [44], vandetanib [45] and aflibercept [46]. For cabozantinib [47], nintedanib [48] and regorafenib [49], heart failure was not mentioned in the SmPCs.

A common frequency of cardiac arrhythmia was reported for bevacizumab and an uncommon frequency for pazopanib, ponatinib and vandetanib, while for eight drugs it was not mentioned. Concerning torsades de pointes/QT prolongation, a very common frequency was reported for vandetanib and a common frequency for Lenvatinib, while it was not mentioned in the SmPC of five AADs: three biological agents, aflibercept, bevacizumab and ramucirumab [50] and two PKIs, axitinib and cabozantinib [51]. As for nintedanib and regorafenib, the frequency was not known.

Regarding ischemic heart disease or myocardial infarction, a common frequency was reported in the SmPC of lenvatinib, ponatinib, sorafenib and sunitinib and an uncommon frequency in the SmPC of cabozantinib, nintedanib, pazopanib and regorafenib. The frequency of ischemic heart disease or myocardial infarction in the SmPC of axitinib, bevacizumab and ramucirumab was not known, and these AEs were not mentioned for aflibercept and vandetanib. The frequency of cerebral hemorrhage was reported only for ponatinib and sunitinib (uncommon). As for cerebral ischemia, a common frequency was reported for bevacizumab, lenvatinib, ponatinib and vandetanib, while an uncommon frequency was reported for cabozantinib, pazopanib and sunitinib.

A very common frequency of embolic and thrombotic events was reported for bevacizumab, while for aflibercept, axitinib, cabozantinib, nintedanib, pazopanib and sunitinib a common frequency was reported in the SmPC. Regorafenib, sorafenib and vandetanib SmPCs do not report the frequency.

Finally, pericardial disease was reported to be common in patients receiving ponatinib and uncommon in patients treated with sunitinib, while it was not mentioned for all other drugs.

Figure 2 reports, for each AAD and CVAE, the frequency found in the SmPC and the SRS signals of disproportionality reporting that emerged from the included studies.

### 2.4. Other Adverse Events Reported in SRS Studies

Eight studies reported disproportionality signals for other non-CVAEs [18,25,28,29,35,36]. Disproportionality values are displayed in Figure S2.

Liao et al. focused on endocrine disorders, especially thyroid dysfunction. In their study, lenvatinib had the highest disproportionality signal of hypothyroidism (ROR = 13.47, 95% CI = 11.54–15.72), followed by sunitinib (ROR = 11.53, 95% CI = 10.60–12.54). The median time of onset of hyperthyroidism for all the VEGFR-TKIs was 32 days (interquartile range (IQR) 14–2100), while for hypothyroidism it was 29 days (IQR 10–184). Meanwhile, lenvatinib had the shortest median time to onset of hypothyroidism (median 12 days, IQR 4–32 days), and ponatinib had the longest time (median 429 days, IQR 46–947 days) [18]. Two studies focused on the osteonecrosis of the jaw [25,35]. Toriumi et al., applying multiple logistic regression model approach, found that sunitinib had a higher risk of osteonecrosis of the jaw, (OR = 9.76, CI 95% = 5.45–17.50) [35], while Zhang et al. found that sorafenib was also significantly associated with this event (OR  =  1.5, CI 95%: 1.2–1.9) [25].

Yoshida et al. assessed the clinical features of hand–foot syndrome (HFS) associated with AADs: they show that lapatinib and regorafenib exhibited a higher reporting ratio and ROR of drug-induced HFS than other drugs [ROR = 130.4, 95% CI = 110.7–153.6 and ROR = 63.3, 95% CI = 55.2–72.6], respectively. A disproportionality signal was also observed for sorafenib (ROR = 29.0, 95% CI = 25.8–32.7), sunitinib (ROR = 13.9, 95% CI = 11.7–16.5), pazopanib (ROR = 6.1, 95% CI = 4.3–8.7) and lenvatinib (ROR = 4.2, 95% CI = 2.8–6.4). Analysis of time-to-onset profiles revealed that the median (interquartile range: 25.0–75.0%) of drug-induced HFS caused by both regorafenib and sorafenib were 9.0 (6.0–14.0) days [36].

In two descriptive analyses, Yang et al. assessed the metabolism and nutrition AEs of antitumor agents, including AADs using VigiBase. Comparing the records of metabolism and nutrition disorders with total retrieved records, sorafenib showed the highest ratio (around 10%) [30]. In a second study, the same authors focused on AEs associated with monoclonal antibody drugs, and bevacizumab was reported to have higher reporting for hyperglycemic records [31].

Wichelmann et al. in a descriptive study, assessed the association between bevacizumab and gastrointestinal perforation. They identified 2874 records of bevacizumab-induced gastrointestinal perforation. The mean age of the patients was 61.9 ± 11.4 years. A total of 698 cases included descriptive locations of perforations with most occurring in the large intestine (385 cases, 55.2% of specifically described cases) [21].

Finally, Minnema et al. assessed the linkage to monoclonal antibodies (mAbs) and neuropsychiatric adverse effects. In their analysis, RORs were estimated for each mAb using bevacizumab as the reference [29]. For depression, the association (relative to bevacizumab) was strongest for natalizumab (ROR = 5.7, 95% CI = 5.0–6.4), followed by belimumab (ROR = 5.1, 95% CI = 4.2–6.2).

## 3. Discussion

Antiangiogenic therapy is a fundamental part of cancer pharmacotherapy, but the side effects of these drugs can compromise the quality of life of oncologic patients. Therefore, close clinical monitoring and early intervention are necessary to minimize the risk of adverse reactions.

This study provided an overview of published studies on AAD-induced side effects collected in the SRS databases as the safety assessment of drugs in clinical trials may have some limitations. First, important safety issues may not be detectable in clinical trials because the frequency of many AEs is too low, and adverse effects of cancer therapies may occur many years after drug administration [52]; second, studies have shown that women and racial/ethnic minorities are deeply underrepresented in clinical trials (and in particular in clinical trials concerning cancer) and the effectiveness and the safety in real-world may be different than expected [53,54,55]. Given the limitations of clinical trials, postmarketing studies play an essential role in assessing the true risk profile of drugs in real-life practice.

In our study, we reported the safety profile of AADs by analyzing the global repositories of safety reports and we found some disproportionality signals for which there is still little information reported in SmPCs.

Arterial aneurysm/aortic dissection was the most studied cardiovascular AE in SRS studies. However, the frequency of these reactions is not known in the SmPCs for all drugs evaluated, except for pazopanib (rare). Large pharmacoepidemiology studies are warranted to clarify this aspect.

As for embolic and thrombotic events, the frequency was reported as common (very common for bevacizumab), while for regorafenib, sorafenib and vandetanib, it was not mentioned in their respective SmPCs. For these three drugs, no pharmacovigilance studies using SRSs were retrieved, implying a possible knowledge gap on the safety of these drugs for these AEs. Moreover, one study reporting a disproportionality signal of pericardial disease was found for axitinib, while this AE was not mentioned in the respective SmPC.

Our study also highlights that SRS studies did not find any disproportionality signal for cardiac arrhythmia for any drugs, which was reported to have a common frequency in the bevacizumab SmPCs.

In the study by Goldman, it was observed that several CVAEs did not exhibit a class effect but were significantly overreported with specific VEGFR-TKIs. For instance, lenvatinib showed at least one disproportionality signal for seven out of the nine CVAEs analyzed.

Despite that CVAEs are generally mentioned for each AAD in SmPC, we highlight that these drugs have a different safety profile that could be related to its molecular structure and pharmacological targets. To antiangiogenic class belong tyrosine kinase inhibitors and monoclonal antibodies, which differ in structure, mechanism of action and metabolism. Notably, other articles focusing on tyrosine kinase inhibitors reported the potential cardiovascular risk with these drugs [16,56].

Many antiangiogenic tyrosine kinase inhibitors are so-called multitargeted kinase inhibitors. These agents target several different kinases, which are involved in several signaling pathways (cabozantinib, lenvatinib, nintedanib, ponatinib, pazopanib, regorafenib, sorafenib, sunitinib, vandetanib). It is reasonable to expect that inhibitors of multiple kinases possess a broader efficacy but also a reduced safety than a single-target inhibitor. In contrast, monoclonal antibodies are not able to pass through the cell membrane so they can only act on molecules expressed on the cell surface (ramucirumab for VEGFR-2) or on secreted molecules (bevacizumab for VEGF-A) [57].

The main molecular mechanism underlying CVAEs of all AADs is endothelial dysfunction [58]. The studies of Wang and Guyon found that VEGF inhibition may cause arterial/aneurism as a class effect of AADs [20,27]. In fact, they showed disproportionality reporting of artery dissections or aneurysms for biological and PKIs agents. Despite that this review only considered AADs used for cancer patients (systemic administration), other studies could highlight the possible cardiovascular safety profile of AADs when given with intraocular injections. We recently highlighted different inflammatory-related intraocular pressure among intravitreal AADs [59], which could potentially influence the systemic bioavailability of AADs in patients with compromised ocular functions [60,61]. Concerning other AEs, a higher ROR signal for HFS was detected in all PKIs compared with bevacizumab (ROR = 63.3, 95% CI = 55.2–72.6 and ROR = 1.6, 95% CI = 1.2–2.1 for regorafenib and bevacizumab, respectively). Everolimus showed a lower incidence of drug-induced HFS than other PKIs (ROR = 0.9, 95% CI = 0.5–1.6), maybe related to the inefficacy of everolimus to inhibit VEGFR. Instead, it reduces the VEGF production, which conveys with bevacizumab activity [62].

Interestingly, in agreement with a meta-analysis on the safety of AADs in the pediatric population, lenvatinib was associated with the most powerful signal of hypothyroidism reports [6,18]. Some further considerations could also be made: the different signals that arose from the pharmacovigilance studies may be due to the database used, the comparator group, the disproportionality methodology applied (Reporting Odds Ratio or Proportional Reporting Ratio), and the confounding factors evaluated. The disproportionality analysis, widely used in pharmacovigilance studies included in this work, focuses exclusively on differences in proportions and some studies did not account for possible confounding effects. Multivariable models analyze multiple variables, with logistic regression, however having been used by some authors [14,24,35]. Finally, only a few studies stratify the results on the basis of age or sex or indication of treatment [16,17,18]. In this regard, this study highlights the importance of conducting postmarketing evaluation of AADs and in particular we believe that the identification of patients who are at risk [63] and those excluded from clinical trials [6] should be carefully monitored.

This study has several strengths: first, to the best of our knowledge, this is the first study that has comprehensively analyzed the safety of AADs by reporting both the frequency of AE from SmPCs and the disproportionality signals that emerged from studies on SRSs. Second, we performed a scoping review using a systematic approach according to PRISMA guidelines for scoping reviews. Finally, this study highlights knowledge gaps about the safety profile of these drugs: (1) several CVAEs have not been mentioned in the SmPC of antiangiogenic drugs and no pharmacovigilance studies were conducted despite a pharmacological rationale on the possible mechanism of such drugs is plausible (i.e., thromboembolic events); (2) despite one study finding a disproportionality signal for axitinib on pericardial disease, this event was not mentioned in the SmPC and further analysis is warranted. However, the authors did not perform a qualitative analysis of the disproportionality signal retrieved and more information is needed for further investigation.

This study has also some limitations. First, we cannot be sure that all the articles present in the literature have been captured by the search strings despite the authors using two literature databases according to PRISMA scoping review guidelines. Second, despite several studies reporting disproportionality signals on anti-VEGF drugs, only a few of them performed further validation of the signals by assessing the quality of reports and the causal relationship of the drug to AEs (two out of the 17 studies that perform disproportionality analyses) [15,32]. Finally, pharmacoepidemiological studies were not considered for inclusion in this review. This missing piece of information may have impaired the comprehensive nature of this study in evaluating the safety profile of antiangiogenic drugs. However, we believe that this scoping review, which focuses on an entire class of drugs and directly relates information from SmPCs and SRSs studies, may be considered a novel approach to highlighting possible drug safety concerns and be considered as a guide for conducting targeted postmarketing surveillance studies of AADs in cardiovascular toxicities.

## 4. Methodology

This scoping review was conducted according to PRISMA guidelines for scoping reviews [63]. A protocol was published on OpenScience framework. (https://osf.io/gtq4e/ (accessed on 30 June 2022).

### 4.1. Literature Search and Eligibility Criteria

PubMed and ISI Web of Knowledge were searched for retrieving the studies of interest. Articles that were published between 1 January 2000 and 30 June 2022 were considered suitable for inclusion. The search strategy is defined in Table S1 of the Supplementary Material for each literature database.

A snowballing search was also conducted to retrieve additional papers of interest by examining the references cited in the included articles. SmPCs of anticancer anti-VEGF drugs were also searched on the European Medicine Agency Website. As known, the SmPC reports the frequency of adverse events (AEs) from clinical trials, postauthorization safety studies and spontaneous reports for which, after careful evaluation, a causal relationship between the drug and the AE is at least a reasonable possibility.

### 4.2. Eligibility Criteria

All studies using SRSs to evaluate safety of antiangiogenic drugs used in patients with tumors were included. Eligible studies had to be written in English and studies with no full text available were excluded.

### 4.3. Study Selection

Two researchers (VC and AS) screened all titles and abstracts of the references retrieved. Potentially relevant studies were further assessed through examination of full texts. The reviewers worked independently, in parallel and blinded to each other. Disagreements between the two reviewers were resolved through discussion with a third author (SD).

### 4.4. Data Extraction

One author extracted the information for the selected studies (VC) and another author validated the extraction (AS). Information was collected in a specific data sheet (Excel). The following information was extracted from the included studies found in PubMed and ISI Web of Science: reference, SRS type (e.g., FAERS, VIGIBASE), number of records screened, type of adverse reaction evaluated (pterm), drugs evaluated, time to onset and outcome (e.g., disproportionality outcomes). As for SmPCs, the following information was extracted: frequency (very common, common, uncommon, rare, or not known). AEs were also considered as not known if they were mentioned in the section of undesirable effects but were not reported in the table of Section 4.8 of the SmPC (only the frequency of AEs for antiangiogenic monotherapy were extracted). Finally, if the SmPC did not report the reaction in the undesirable effects section, it was considered as “not mentioned”.

## 5. Conclusions

The studies included in this work highlight the importance of monitoring and managing adverse events associated with antiangiogenic cancer therapies, particularly cardiovascular toxicities. Despite a higher risk of incurring cardiovascular adverse events for each drug given their mechanism of action, we know that these drugs are different in terms on their structure, pharmacokinetic and pharmacodynamic profiles and their respective cardiovascular safety is quite different among them. Bevacizumab was found to have the highest reporting odds ratio for adverse cardiotoxicity, including pericardial diseases. Aneurysm/aortic dissection events were also identified as potential serious adverse events associated with some antiangiogenic inhibitors. This study also highlights for which drugs there are still few or no information about their safety profile. Overall, the findings emphasize the need for continued research and vigilance in the monitoring and management of adverse events associated with antiangiogenic cancer therapies.

## Figures and Tables

**Table 1:** Pharmacological properties of antiangiogenic drugs grouped by class.

Pharmaceutical Class	Drug	Category	Target
Biological	Aflibercept	Soluble recombinant fusion protein	VEGF A-D, PlGF
Biological	Bevacizumab	Humanized monoclonal antibody	VEGF-A
Biological	Ramucirumab	Humanized monoclonal antibody	VEGFR-2
Small molecule	Axitinib	PKIs	VEGFR-1/2/3
Small molecule	Cabozantinib	PKIs	VEGFR-2, c-Met, ROS1, TYRO3, MER, Ret, Kit, TRKB, Flt-3, AXL, Tie-2
Small molecule	Lenvatinib	PKIs	VEGFR-1/2/3, FGFRs, PDGFR-α, c-Kit, RET
Small molecule	Nintedanib	PKIs	VEGFR-1/2/3, FGFR-1/2, PDGFR-α/β
Small molecule	Pazopanib	PKIs	VEGFR-1/2/3, PDGFR-α/β, c-Kit
Small molecule	Ponatinib	PKIs	VEGFR, SRC, ABL, FGFR, PDGFR
Small molecule	Regorafenib	PKIs	VEGFR-1/2/3, FGFR-1/2, PDGFR-α, Tie-2, RAF-1, BRAF, BRAF^V600E^, c-Kit receptor
Small molecule	Sorafenib	PKIs	VEGFR-1/2/3, PDGFR-β, Raf serine/threonine kinases, c-Kit receptor
Small molecule	Sunitinib	PKIs	VEGFR-1/2/3, PDGFR-α, c-Kit receptor, RET, FLT3, CSF-1R
Small molecule	Vandetanib	PKIs	VEGFR-2, EGFR, RET

**Table 2:** Characteristics of included studies.

Reference	SRSs Type	Total Number of Anti-VEGF Record	Study Period	Systemic Antiangiogenic Drugs Studied	Adverse Events	Disease	Type of Analysis	Comparison Groups	Outcome
Bai et al., 2021 [14]	FAERS	409	2013–2019	Bevacizumab + PD-(L)1	No restriction	Nonsmall cell lung cancer	Logistic regression	PD-(L)1 monotherapy vs. PD-(L)1 + bevacizumab	Odds ratio
Cheng et al., 2021 [15]	FAERS	240	ns	VEGF inhibitors	Arterial aneurysm/dissection	Cancer	Descriptive	-	-
Cirmi et al., 2020 [16]	FAERS	3101	April 2008–December 2008	Ponatinib	Cardiovascular toxicities	Cancer	Disproportionality analysis	TKIs vs. other anticancer drugs	Reporting odds ratio
Clapes et al., 2018 [32]	French pharmacovigilance database (regional)	49	2003–2015	Sorafenib Sunitinib	No restriction	Cancer	Descriptive	-	-
Cutroneo et al., 2017 [37]	Italian spontaneous reporting system	2173	2005–2016	Aflibercept Bevacizumab	No restriction	Cancer and retinal diseases	Disproportionality analysis	Anti-VEGF vs. Other suspected drugs	Proportional reporting ratio
De Campaigno et al., 2017 [26]	VigiBase	45,832	2001–2015	Axitinib Pazopanib Sorafenib Sunitinib Vandetanib	Cardiac failure	Cancer	Disproportionality analysis	Protein kinase inhibitor vs. Other protein kinase inhibitors	Reporting odds ratio
Egron et al., 2014 [33]	French pharmacovigilance database	271	2008–2009	Sorafenib Sunitinib	No restriction	Cancer	Preventability	-	French ADR preventability scale score
Goldman et al., 2021 [17]	FAERS	51,836	2014–2019	Axitinib Cabozatinib Lenvatinib Pazopanib Sorafenib Sunitinib	Cardiovascular toxicities	Cancer and other	Disproportionality analysis	Anti-VEGF TKIs vs. other drugs in the full database	Reporting odds ratio and Information component
Gouverneur et al., 2017 [28]	VigiBase	13,920	Until December 2016	Aflibercept Bevacizumab Regorafenib	No restriction	Metastatic colorectal cancer	Disproportionality analysis	Target therapy vs. all other anticancer drugs	Proportional reporting ratio
Guyon et al., 2021 [27]	VigiBase	494	2005–2019	Axitinib Bevacizumab Cabozatinib Lenvatinib Nintedanib Pazopanib Ramucirumab Sorafenib Sunitinib	Arterial aneurysm/dissection	Cancer	Disproportionality analysis	Antiangiogenic drugs vs. other anticancer drugs	Proportional reporting ratio and information component
Liao et al., 2021 [18]	FAERS	1567	2004–2020	Axitinib Cabozatinib Lenvatinib Pazopanib Ponatinib Regorafenib Sorafenib Sunitinib Vandetanib	Thyroid dysfunction	ns	Disproportionality analysis	VEGFR-TKIs vs. all other drugs	Reporting odds ratio, Proportional reporting ratio, information component and empirical Bayesian geometric mean
Makunts et al., 2021 [19]	FAERS	20,062 (ICI)		Axitinib + pembrolizumab or avelumab	Myocarditis	Cancer	Disproportionality analysis	ICI axitinib or ICI alone or ICI combinations vs. chemotherapy	Reporting odds ratio
Minnema et al., 2019 [29]	VigiBase	9455 (depression), 1770 (suicidal ideation and behavior)	Until December 2017	Bevacizumab Ramucirumab	Depression and suicidal ideation and behavior	Cancer and other	Disproportionality analysis	mABs vs. bevacizumab	Reporting odds ratio
Taugourdeau-Raymond et al., 2012 [34]	French pharmacovigilance database	455	2005–2010	Bevacizumab	No restriction	Cancer	Descriptive	-	-
Toriumi et al., 2020 [35]	JADER	4597	2004–2019	Sunitinib	Osteonecrosis of the jaw	ns	Disproportionality analysis and logistic regression	Suspected drugs vs. all other drugs	Reporting odds ratio and odds ratio
Wang et al., 2021 [20]	FAERS	634	2004–2020	Aflibercept Axitinib Bevacizumab Cabozatinib Lenvatinib Nintedanib Pazopanib Ponatinib Ramucirumab Regorafenib Sorafenib Sunitinib Vandetanib	Arterial aneurysm/dissection	Cancer	Disproportionality analysis	Suspected drugs vs. all other drugs	Reporting odds ratio
Wichelmann et al., 2021 [21]	FAERS	2874	2004–2021	Bevacizumab	Gastrointestinal perforation	Cancer	Descriptive		-
Wittayanukorn et al., 2017 [22]	FAERS	167	2004–2012	Bevacizumab	Cardiotoxicity	Breast cancer	Disproportionality analysis	Target therapy vs. all other drugs	Reporting odds ratio
Yagi et al., 2021 [23]	FAERS	1520	2010–2015	Bevacizumab	Hypertension	ns	Disproportionality analysis	Bevacizumab vs. other than bevacizumab	Reporting odds ratio
Yang et al., 2017 [30]	VigiBase	ns	Until December 2016	Sorafenib	Glycaemic Adverse Drug Reactions	Pancreatic Cancer	Descriptive		-
Yang et al., 2022 [31]	VigiBase	ns	Until 2019	Bevacizumab	Blood glucose related adverse drug reaction	Cancer	Descriptive		-
Ye et al., 2021 [24]	FAERS	23,067	2014–2019	Axitinib Cabozatinib Lenvatinib Nintedanib Pazopanib Regorafenib Sorafenib Sunitinib	cardiac arrhythmia	Cancer	Disproportionality analysis and logistical regression	Protein kinase inhibitors vs. non-Protein kinase inhibitors	Reporting odds ratio and odds ratio
Yoshida et al., 2022 [36]	JADER	665	2004–2020	Axitinib Bevacizumab Lenvatinib Pazopanib Regorafenib Sunitinib Sorafenib	hand–foot syndrome	Cancer	Disproportionality analysis	Suspected drugs vs. all other drugs	Reporting odds ratio
Zhang et al., 2016 [25]	FAERS	1230	2010–2014	Axitinib Bevacizumab Pazopanib Sunitinib Sorafenib	Osteonecrosis of the jaw	Cancer or osteoporosis	Disproportionality analysis	Suspected drugs vs. all other drugs	Reporting odds ratio

FAERS: FDA adverse events reporting system; JADER: Japanese adverse drug report (database); Ns: not specified; SRSs: Spontaneous reporting systems.

## Data Availability

Data is contained within the article.

## Supplementary Materials

The following supporting information can be downloaded at: https://www.mdpi.com/article/10.3390/ph16060867/s1, Table S1: Search strategy. Figure S1: Summary of disproportion analysis for drugs included in the study. Figure S2: Other Adverse events reported in SRSs studies.
