# Supplementary material for: Uncovering Knowledge Gaps in the Safety Profile of Antiangiogenic Drugs in Cancer Patients: Insights from Spontaneous Reporting Systems Studies
Source: Pharmaceuticals (Basel). 2023 Jun 12;16(6):867. doi: 10.3390/ph16060867 (PMC10302288; doi:10.3390/ph16060867)
Supplement: Supplementary file 1 [file pharmaceuticals-16-00867-s001.zip › pharmaceuticals-2423059-supplementary.pdf]

Supplementary material

Table S1. Search strategy

PUBMED
(anti-VEGF or "antiangiogenic" or “anti-angiogenic” or bevacizumab or sorafenib or sunitinib or cabozatinib or propranolol or axitinib or aflibercept or dovitinib or lenvatininb or nintedanib or pazopanib or ponatinib or ramucirumab or regorafenib or rivoceranib or tivozatinib or trebananib or vandetanib) AND (pharmacovigilance OR “spontaneous reporting system” OR pharmacovigilance[mesh] or pharmaco-vigilance OR vigibase OR faers OR eudravigilance OR “spontaneous reporting”[title/abstract] OR “case/non-case”) AND (Tumor or cancer or tumors or cancers or tumor[mesh]) NOT (“Phase I”[title] OR “Phase 1”[title] OR “Phase II”[title] OR “Phase III”[title] OR “Phase 2”[title] OR “Phase 3”[title] OR “clinical trial”[publication type])
WEB OF KNOWLEDGE
All fields: anti-VEGF or "antiangiogenic" or “anti-angiogenic” or bevacizumab or sorafenib or sunitinib or cabozatinib or propranolol or axitinib or aflibercept or dovitinib or lenvatininb or nintedanib or pazopanib or ponatinib or ramucirumab or regorafenib or rivoceranib or tivozatinib or trebananib or vandetanib<br>AND all fields: pharmacovigilance OR “spontaneous reporting system” OR pharmaco-vigilance OR vigibase OR faers OR eudravigilance OR “spontaneous reporting” OR “case/non-case”<br>AND all fields: tumor or cancer or tumors or cancers<br>NOT title: “Phase I” or “Phase 1” OR “Phase II” OR “Phase III” OR “Phase 2” OR “Phase 3”

Figure S1
Summary of disproportion analysis for drugs included in the study

Reference	ADR	Database	Drug	Type of analysis	Disproportionality value	95% CI LOW	95% CI UP	Time to onset (days)	Range		
Cheng et al., 2021	Arterial aneurism/dissection	FAERS	Axitinib		Descriptive			92	9	1564	
Cheng et al., 2021	Arterial aneurism/dissection	FAERS	Bevacizumab		Descriptive			97	1	1955	
Cheng et al., 2021	Arterial aneurism/dissection	FAERS	Cabozantinib		Descriptive			38	N/A		
Cheng et al., 2021	Arterial aneurism/dissection	FAERS	Lenvatinib		Descriptive			82	1	371	
Cheng et al., 2021	Arterial aneurism/dissection	FAERS	Pazopanib		Descriptive			57	26	95	
Cheng et al., 2021	Arterial aneurism/dissection	FAERS	Ponatinib		Descriptive			220	5	616	
Cheng et al., 2021	Arterial aneurism/dissection	FAERS	Ramucirumab		Descriptive			60	2	260	
Cheng et al., 2021	Arterial aneurism/dissection	FAERS	Regorafenib		Descriptive			40	14	117	
Cheng et al., 2021	Arterial aneurism/dissection	FAERS	Sorafenib		Descriptive			81	15	913	
Cheng et al., 2021	Arterial aneurism/dissection	FAERS	Sunitinib		Descriptive			166	7	1416	
Cheng et al., 2021	Arterial aneurism/dissection	FAERS	Vandetanib		Descriptive			1	N/A		
Cheng et al., 2021	Arterial aneurism/dissection	FAERS	ziv-aflibercept		Descriptive			21	14	216	
Goldman et al., 2021	Arterial aneurism/dissection	FAERS	Axitinib	ROR	4.47	2.13	9.4				
Goldman et al., 2021	Arterial aneurism/dissection	FAERS	Cabozantinib	ROR	0.27	0.04	1.9				
Goldman et al., 2021	Arterial aneurism/dissection	FAERS	Lenvatinib	ROR	7.14	4.22	12.08				
Goldman et al., 2021	Arterial aneurism/dissection	FAERS	Pazopanib	ROR	1.12	0.5	2.51				
Goldman et al., 2021	Arterial aneurism/dissection	FAERS	Sunitinib	ROR	6.16	4.37	8.7	95*	22.5	366	*not specified for single drug
Guyon et al., 2021	Arterial aneurism/dissection	VigiBase	Axitinib	PRR	2.52	1.51	4.19	NR	NR	NR	
Guyon et al., 2021	Arterial aneurism/dissection	VigiBase	Bevacizumab	PRR	4.08	3.54	4.7	NR	NR	NR	
Guyon et al., 2021	Arterial aneurism/dissection	VigiBase	Cabozantinib	PRR	2.25	1.24	4.07	NR	NR	NR	
Guyon et al., 2021	Arterial aneurism/dissection	VigiBase	Lenvatinib	PRR	4.17	2.3	7.56	NR	NR	NR	
Guyon et al., 2021	Arterial aneurism/dissection	VigiBase	Nintedanib	PRR	2.08	1.27	3.4	NR	NR	NR	
Guyon et al., 2021	Arterial aneurism/dissection	VigiBase	Pazopanib	PRR	1.57	1.11	2.23	NR	NR	NR	
Guyon et al., 2021	Arterial aneurism/dissection	VigiBase	Ramucirumab	PRR	3.34	1.89	5.9	NR	NR	NR	
Guyon et al., 2021	Arterial aneurism/dissection	VigiBase	Sunitinib	PRR	2.38	1.87	3.01	NR	NR	NR	
Wang et al., 2021	Arterial aneurism/dissection	FAERS	Aflibercept	ROR	1.21	0.86	1.7	NR	NR	NR	
Wang et al., 2021	Arterial aneurism/dissection	FAERS	Axitinib	ROR	1.25	0.74	2.11	NR	NR	NR	
Wang et al., 2021	Arterial aneurism/dissection	FAERS	Bevacizumab	ROR	3.05	2.67	3.48	NR	NR	NR	
Wang et al., 2021	Arterial aneurism/dissection	FAERS	Cabozantinib	ROR	0.61	0.34	1.07	NR	NR	NR	
Wang et al., 2021	Arterial aneurism/dissection	FAERS	Lenvatinib	ROR	3	2.11	4.28	NR	NR	NR	
Wang et al., 2021	Arterial aneurism/dissection	FAERS	Nintedanib	ROR	2.12	1.43	3.14	NR	NR	NR	
Wang et al., 2021	Arterial aneurism/dissection	FAERS	Pazopanib	ROR	0.61	0.39	0.95	NR	NR	NR	
Wang et al., 2021	Arterial aneurism/dissection	FAERS	Pegaptanib	ROR	1.49	0.21	10.64	NR	NR	NR	
Wang et al., 2021	Arterial aneurism/dissection	FAERS	Ponatinib	ROR	2.03	1.01	4.06	NR	NR	NR	
Wang et al., 2021	Arterial aneurism/dissection	FAERS	Ramucirumab	ROR	3.68	2.18	6.23	NR	NR	NR	
Wang et al., 2021	Arterial aneurism/dissection	FAERS	Regorafenib	ROR	0.79	0.39	1.57	NR	NR	NR	
Wang et al., 2021	Arterial aneurism/dissection	FAERS	Sorafenib	ROR	1.41	1.02	1.93	NR	NR	NR	
Wang et al., 2021	Arterial aneurism/dissection	FAERS	Sunitinib	ROR	1.93	1.59	2.36	NR	NR	NR	
Wang et al., 2021	Arterial aneurism/dissection	FAERS	Vandetanib	ROR	1.57	0.39	6.29	NR	NR	NR	
Cirmi et al., 2021	Cardiac failure	FAERS	Ponatinib	ROR	1.8	1.4	2.4	48	13	199	
Cutroneo et al., 2017	Cardiac failure	Italian spontaneous reporting system	Bevacizumab	PRR	4.69	3.24	6.79	NR	NR	NR	
De Campaigno et al., 2017	Cardiac failure	VigiBase	Sunitinib	ROR	1.67	1.51	1.84	NR	NR	NR	
Goldman et al., 2021	Cardiac failure	FAERS	Axitinib	ROR	1.09	0.85	1.4	NR	NR	NR	
Goldman et al., 2021	Cardiac failure	FAERS	Cabozantinib	ROR	0.7	0.57	0.85	NR	NR	NR	
Goldman et al., 2021	Cardiac failure	FAERS	Lenvatinib	ROR	2.21	1.5	3.27	NR	NR	NR	
Goldman et al., 2021	Cardiac failure	FAERS	Pazopanib	ROR	1.1	0.96	1.26	NR	NR	NR	
Goldman et al., 2021	Cardiac failure	FAERS	Sunitinib	ROR	2.01	1.68	2.4	NR	NR	NR	
Gouverneur et al., 2017	Cardiac failure	VigiBase	Aflibercept (<75 anni)	PRR	1.5	0.81	2.78	NR	NR	NR	
Gouverneur et al., 2017	Cardiac failure	VigiBase	Aflibercept (>75 anni)	PRR	not reached (less of three serious cases)			NR	NR	NR	
Gouverneur et al., 2017	Cardiac failure	VigiBase	Bevacizumab (<75 anni)	PRR	0.65	0.51	0.83	NR	NR	NR	
Gouverneur et al., 2017	Cardiac failure	VigiBase	Bevacizumab (>75 anni)	PRR	0.77	0.53	1.13	NR	NR	NR	
Gouverneur et al., 2017	Cardiac failure	VigiBase	Regorafenib (<75 anni)	PRR	0.29	0.16	0.52	NR	NR	NR	
Gouverneur et al., 2017	Cardiac failure	VigiBase	Regorafenib (>75 anni)	PRR	0.66	0.34	1.26	NR	NR	NR	
Cirmi et al., 2021	Cardiac arrhythmia	FAERS	Ponatinib	ROR	1	0.5	2.2	NR	NR	NR	
Cutroneo et al., 2017	Cardiac arrhythmia	Italian spontaneous reporting system	Bevacizumab	PRR	0.75	0.45	1.27	NR	NR	NR	
Ye et al., 2021	Cardiac arrhythmia	FAERS	Axitinib	ROR	1.12	0.76	1.63	NR	NR	NR	
Ye et al., 2021	Cardiac arrhythmia	FAERS	Cabozantinib	ROR	0.74	0.48	1.14	NR	NR	NR	
Ye et al., 2021	Cardiac arrhythmia	FAERS	Lenvatinib	ROR	0.98	0.68	1.4	NR	NR	NR	
Ye et al., 2021	Cardiac arrhythmia	FAERS	Nintedanib	ROR	0.61	0.48	0.77	NR	NR	NR	
Ye et al., 2021	Cardiac arrhythmia	FAERS	Pazopanib	ROR	0.68	0.47	0.97	NR	NR	NR	
Ye et al., 2021	Cardiac arrhythmia	FAERS	Regorafenib	ROR	1.3	0.9	1.87	NR	NR	NR	
Ye et al., 2021	Cardiac arrhythmia	FAERS	Sorafenib	ROR	0.62	0.4	0.96	NR	NR	NR	
Ye et al., 2021	Cardiac arrhythmia	FAERS	Sunitinib	ROR	0.55	0.41	0.72	NR	NR	NR	
Ye et al., 2021	Cardiac arrhythmia	FAERS	Ponatinib	ROR	3.03	2.21	4.14	NR	NR	NR	
Wittayanukorn et al., 2015	Cardiotoxicity	FAERS	Bevacizumab	ROR	3.17	2.7	3.71	NR	NR	NR	
Wittayanukorn et al., 2015	Cardiotoxicity	FAERS	Lapatinib	ROR	1.2	1.01	1.41	NR	NR	NR	
Wittayanukorn et al., 2015	Cardiotoxicity	FAERS	Bevacizumab + Cycloph	ROR	6.71	4.95	9.1	NR	NR	NR	
Wittayanukorn et al., 2015	Cardiotoxicity	FAERS	Bevacizumab + Doxo	ROR	8.76	6.3	12.18	NR	NR	NR	
Cirmi et al., 2021	Cardiomyopathy	FAERS	Ponatinib	ROR	0.6	0.3	1.2	NR	NR	NR	
Cutroneo et al., 2017	Cardiomyopathy	Italian spontaneous reporting system	Bevacizumab	PRR	4.74	2.24	10.04	NR	NR	NR	
Goldman et al., 2021	Cardiomyopathy	FAERS	Axitinib	ROR	1.7	0.71	4.09	NR	NR	NR	
Goldman et al., 2021	Cardiomyopathy	FAERS	Cabozantinib	ROR	1.04	0.5	2.19	NR	NR	NR	
Goldman et al., 2021	Cardiomyopathy	FAERS	Lenvatinib	ROR	1.63	0.73	3.63	NR	NR	NR	
Goldman et al., 2021	Cardiomyopathy	FAERS	Pazopanib	ROR	2.51	1.68	3.75	NR	NR	NR	
Goldman et al., 2021	Cardiomyopathy	FAERS	Sunitinib	ROR	1.81	1.14	2.88	NR	NR	NR	
Cirmi et al., 2021	Ischemic heart disease	FAERS	Ponatinib	ROR	2.9	2.4	3.5	280.5	75	365	
Cutroneo et al., 2017	Ischemic heart disease	Italian spontaneous reporting system	Bevacizumab	PRR	5.63	3.98	7.96	NR	NR	NR	
Goldman et al., 2021	Ischemic heart disease	FAERS	Axitinib	ROR	1.03	0.82	1.28	NR	NR	NR	
Goldman et al., 2021	Ischemic heart disease	FAERS	Cabozantinib	ROR	0.51	0.41	0.62	NR	NR	NR	
Goldman et al., 2021	Ischemic heart disease	FAERS	Lenvatinib	ROR	1.41	1.19	1.67	NR	NR	NR	
Goldman et al., 2021	Ischemic heart disease	FAERS	Pazopanib	ROR	0.66	0.56	0.76	NR	NR	NR	
Goldman et al., 2021	Ischemic heart disease	FAERS	Sunitinib	ROR	0.74	0.64	0.85	NR	NR	NR	
Gouverneur et al., 2017	Ischemic heart disease	VigiBase	Aflibercept (<75 anni)	PRR	0.61	0.34	1.1	NR	NR	NR	
Gouverneur et al., 2017	Ischemic heart disease	VigiBase	Aflibercept (>75 anni)	PRR	1.42	0.54	3.69	NR	NR	NR	
Gouverneur et al., 2017	Ischemic heart disease	VigiBase	Bevacizumab (<75 anni)	PRR	0.82	0.72	0.94	NR	NR	NR	
Gouverneur et al., 2017	Ischemic heart disease	VigiBase	Bevacizumab (>75 anni)	PRR	1.12	0.86	1.47	NR	NR	NR	
Gouverneur et al., 2017	Ischemic heart disease	VigiBase	Regorafenib (<75 anni)	PRR	0.83	0.68	1.03	NR	NR	NR	
Gouverneur et al., 2017	Ischemic heart disease	VigiBase	Regorafenib (>75 anni)	PRR	0.78	0.47	1.31	NR	NR	NR	
Guyon et al., 2021	Cerebral aneurysm	VigiBase	Axitinib	PRR	2.52	1.04	6.08	NR	NR	NR	
Guyon et al., 2021	Cerebral aneurysm	VigiBase	Bevacizumab	PRR	3.76	2.92	4.84	NR	NR	NR	
Guyon et al., 2021	Cerebral aneurysm	VigiBase	Sunitinib	PRR	1.89	1.19	2.99	NR	NR	NR	
Goldman et al., 2021	Cerebral hemorrhages	FAERS	Axitinib	ORR	1.15	0.8	1.65	NR	NR	NR	
Goldman et al., 2021	Cerebral hemorrhages	FAERS	Cabozantinib	ORR	0.62	0.45	0.86	NR	NR	NR	
Goldman et al., 2021	Cerebral hemorrhages	FAERS	Lenvatinib	ORR	3.32	2.74	4.02	NR	NR	NR	
Goldman et al., 2021	Cerebral hemorrhages	FAERS	Pazopanib	ORR	0.85	0.67	1.07	NR	NR	NR	
Goldman et al., 2021	Cerebral hemorrhages	FAERS	Sunitinib	ORR	0.79	0.63	1	NR	NR	NR	
Goldman et al., 2021	Cerebral ischemia	FAERS	Axitinib	ROR	1.53	1.25	1.88	NR	NR	NR	
Goldman et al., 2021	Cerebral ischemia	FAERS	Cabozantinib	ROR	0.81	0.67	0.97	NR	NR	NR	
Goldman et al., 2021	Cerebral ischemia	FAERS	Lenvatinib	ROR	2.19	1.87	2.55	NR	NR	NR	
Goldman et al., 2021	Cerebral ischemia	FAERS	Pazopanib	ROR	0.83	0.72	0.97	NR	NR	NR	
Goldman et al., 2021	Cerebral ischemia	FAERS	Sunitinib	ROR	0.81	0.69	0.94	NR	NR	NR	
Cutroneo et al., 2017	CNS vascular disorders	Italian spontaneous reporting system	Bevacizumab	PRR	2.44	1.87	3.2	NR	NR	NR	
Cirmi et al., 2021	Embolic and thrombotic events	FAERS	Ponatinib	ROR	1.4	1.2	1.6	NR	NR	NR	
Cutroneo et al., 2017	Embolic and thrombotic events	Italian spontaneous reporting system	Aflibercept	PRR	4.38	2.01	9.57	NR	NR	NR	
Cutroneo et al., 2017	Embolic and thrombotic events	Italian spontaneous reporting system	Bevacizumab	PRR	15.18	13.78	16.73	NR	NR	NR	
Goldman et al., 2021	Arterial embolic events	FAERS	Axitinib	ROR	0.67	0.32	1.42	NR	NR	NR	
Goldman et al., 2021	Arterial embolic events	FAERS	Cabozantinib	ROR	0.63	0.38	1.05	NR	NR	NR	
Goldman et al., 2021	Arterial embolic events	FAERS	Lenvatinib	ROR	1.23	0.75	2.01	NR	NR	NR	
Goldman et al., 2021	Arterial embolic events	FAERS	Pazopanib	ROR	0.83	0.57	1.2	NR	NR	NR	
Goldman et al., 2021	Arterial embolic events	FAERS	Sunitinib	ROR	1.34	1.01	1.79	NR	NR	NR	
Goldman et al., 2021	Venous embolic events	FAERS	Axitinib	ROR	0.85	0.61	1.19	NR	NR	NR	
Goldman et al., 2021	Venous embolic events	FAERS	Cabozantinib	ROR	1.78	1.52	2.07	NR	NR	NR	
Goldman et al., 2021	Venous embolic events	FAERS	Lenvatinib	ROR	2.79	2.36	3.3	NR	NR	NR	
Goldman et al., 2021	Venous embolic events	FAERS	Pazopanib	ROR	0.69	0.57	0.85	NR	NR	NR	
Goldman et al., 2021	Venous embolic events	FAERS	Sunitinib	ROR	1.19	1.02	1.39	NR	NR	NR	
Cirmi et al., 2021	Hypertension	FAERS	Ponatinib	ROR	3.5	2.9	4.3	NR	NR	NR	
Cutroneo et al., 2017	Hypertension	Italian spontaneous reporting system	Aflibercept	PRR	10.09	6.39	15.95	NR	NR	NR	
Cutroneo et al., 2017	Hypertension	Italian spontaneous reporting system	Bevacizumab	PRR	11.14	10.01	12.39	NR	NR	NR	
Goldman et al., 2021	Hypertension	FAERS	Lenvatinib	ROR	16.74	15.7	17.7	NR	NR	NR	
Goldman et al., 2021	Hypertension	FAERS	Axitinib	ROR	6.5	5.93	7.13	NR	NR	NR	
Goldman et al., 2021	Hypertension	FAERS	Sunitinib	ROR	5.75	6.45	6.06	NR	NR	NR	
Goldman et al., 2021	Hypertension	FAERS	Pazopanib	ROR	4.49	4.23	4.77	NR	NR	NR	
Goldman et al., 2021	Hypertension	FAERS	Cabozantinib	ROR	4.21	3.91	4.53	NR	NR	NR	
Yagi et al., 2020	Hypertension	FAERS	Bevacizumab	ROR	2.45	2.33	2.59	NR	NR	NR	
Yagi et al., 2020	Hypertension	FAERS	Bevacizumab male	ROR	1.66	1.51	1.84	NR	NR	NR	
Yagi et al., 2020	Hypertension	FAERS	Bevacizumab female	ROR	2.38	2.21	2.56	NR	NR	NR	
Yagi et al., 2020	Hypertension	FAERS	Bevacizumab <40	ROR	3.19	2.46	4.14	NR	NR	NR	
Yagi et al., 2020	Hypertension	FAERS	Bevacizumab 40-49	ROR	2.22	1.82	2.71	NR	NR	NR	
Yagi et al., 2020	Hypertension	FAERS	Bevacizumab 50-59	ROR	1.95	1.7	2.24	NR	NR	NR	
Yagi et al., 2020	Hypertension	FAERS	Bevacizumab 60-69	ROR	1.86	1.65	2.09	NR	NR	NR	
Yagi et al., 2020	Hypertension	FAERS	Bevacizumab 70-100	ROR	1.86	1.65	2.12	NR	NR	NR	
Cirmi et al., 2021	Pulmonary Hypertension	FAERS	Ponatinib	ROR	1.3	0.6	3.9	NR	NR	NR	
Cutroneo et al., 2017	Pulmonary Hypertension	Italian spontaneous reporting system	Bevacizumab	PRR	14.76	6.83	31.92	NR	NR	NR	
Goldman et al., 2021	Pulmonary Hypertension	FAERS	Axitinib	ROR	0.08	0.01	0.59	NR	NR	NR	
Goldman et al., 2021	Pulmonary Hypertension	FAERS	Cabozantinib	ROR	0.18	0.07	0.43	NR	NR	NR	
Goldman et al., 2021	Pulmonary Hypertension	FAERS	Lenvatinib	ROR	0.26	0.1	0.7	NR	NR	NR	
Goldman et al., 2021	Pulmonary Hypertension	FAERS	Pazopanib	ROR	0.23	0.12	0.44	NR	NR	NR	
Goldman et al., 2021	Pulmonary Hypertension	FAERS	Sunitinib	ROR	0.27	0.15	0.48	NR	NR	NR	
Cirmi et al., 2021	Torsades de pointes/QT prolongatior	FAERS	Ponatinib	ROR	0.9	0.3	2.4	NR	NR	NR	
Goldman et al., 2021	Torsades de pointes/QT prolongatior	FAERS	Axitinib	ROR	0.45	0.17	1.19	NR	NR	NR	
Goldman et al., 2021	Torsades de pointes/QT prolongatior	FAERS	Cabozantinib	ROR	0.39	0.2	0.78	NR	NR	NR	
Goldman et al., 2021	Torsades de pointes/QT prolongatior	FAERS	Lenvatinib	ROR	2.15	1.44	3.21	NR	NR	NR	
Goldman et al., 2021	Torsades de pointes/QT prolongatior	FAERS	Pazopanib	ROR	0.45	0.26	0.77	NR	NR	NR	
Goldman et al., 2021	Torsades de pointes/QT prolongatior	FAERS	Sunitinib	ROR	0.83	0.56	1.23	NR	NR	NR	
Goldman et al., 2021	Pericardial disease	FAERS	Axitinib	ROR	2.15	1.33	3.46				
Goldman et al., 2021	Pericardial disease	FAERS	Cabozantinib	ROR	1.38	0.93	2.04				
Goldman et al., 2021	Pericardial disease	FAERS	Lenvatinib	ROR	1.2	0.68	2.12				
Goldman et al., 2021	Pericardial disease	FAERS	Pazopanib	ROR	1.27	0.9	1.79				
Goldman et al., 2021	Pericardial disease	FAERS	Sunitinib	ROR	3.15	2.54	3.91	69.5	20.3	225.3	*not specified for single drug
Gouverneur et al., 2017	ntricular arrhythmias and cardiac arr	VigiBase	Bevacizumab (<75 anni)	PRR	1.09	0.88	1.35	NR	NR	NR	
Gouverneur et al., 2017	ntricular arrhythmias and cardiac arr	VigiBase	Bevacizumab (>75 anni)	PRR	1.22	0.77	1.91	NR	NR	NR	
Gouverneur et al., 2017	ntricular arrhythmias and cardiac arr	VigiBase	Aflibercept (<75 anni)	PRR	0.73	0.28	1.94	NR	NR	NR	
Gouverneur et al., 2017	ntricular arrhythmias and cardiac arr	VigiBase	Aflibercept (>75 anni)	PRR	not reached (less of three serious cases)			NR	NR	NR	
Gouverneur et al., 2017	ntricular arrhythmias and cardiac arr	VigiBase	Regorafenib (<75 anni)	PRR	0.19	0.09	0.42	NR	NR	NR	
Gouverneur et al., 2017	ntricular arrhythmias and cardiac arr	VigiBase	Regorafenib (>75 anni)	PRR	not reached (less of three serious cases)			NR	NR	NR	
Makunts et al., 2021	Myocarditis	FAERS	Avelumab+axitinib	ROR	55.6	13.4	222.3	NR	NR	NR	
Makunts et al., 2021	Myocarditis	FAERS	Pembrolizumab+axitinib	ROR	36.9	11.8	115.9	NR	NR	NR	
Makunts et al., 2021	Myocarditis	FAERS	Avelumab	ROR	16.6	4.1	66.8	NR	NR	NR	
Makunts et al., 2021	Myocarditis	FAERS	Pembrolizumab	ROR	24.1	19.7	29.4	NR	NR	NR	

Figure S2

Other Adverse events reported in SRSs studies

Reference	ADR	Database	Drug	Type of analysis	Disproportionality value	95% CI LOW	95% CI UP	Time to onset (days)	Range	
Yoshida et al., 2022	Hand-foot syndrome	JADER	Axitinib	ROR	14.9	11.1	20.1	NR	NR	NR
Yoshida et al., 2022	Hand-foot syndrome	JADER	Bevacizumab	ROR	1.6	1.2	2.1	NR	NR	NR
Yoshida et al., 2022	Hand-foot syndrome	JADER	Lapatinib	ROR	130.4	110.7	153.6	NR	NR	NR
Yoshida et al., 2022	Hand-foot syndrome	JADER	Lenvatinib	ROR	4.2	2.8	6.4	NR	NR	NR
Yoshida et al., 2022	Hand-foot syndrome	JADER	Pazopanib	ROR	6.1	4.3	8.7	NR	NR	NR
Yoshida et al., 2022	Hand-foot syndrome	JADER	Regorafenib	ROR	63.3	55.2	72.6	9	6	14
Yoshida et al., 2022	Hand-foot syndrome	JADER	Sorafenib	ROR	29	25.8	32.7	9	6	14
Yoshida et al., 2022	Hand-foot syndrome	JADER	Sunitinib	ROR	13.9	11.7	16.5	NR	NR	NR
Zhang et al., 2015	Osteonecrosis of the jaw	FAERS	Axitinib	ROR	0.8	0.4	1.5	NR	NR	NR
Zhang et al., 2015	Osteonecrosis of the jaw	FAERS	Bevacizumab	ROR	4.5	4.2	4.9	NR	NR	NR
Zhang et al., 2015	Osteonecrosis of the jaw	FAERS	Pazopanib	ROR	1.3	0.7	2.5	NR	NR	NR
Zhang et al., 2015	Osteonecrosis of the jaw	FAERS	Sorafenib	ROR	1.5	1.2	1.9	NR	NR	NR
Zhang et al., 2015	Osteonecrosis of the jaw	FAERS	Sunitinib	ROR	4.6	4.2	5.1	NR	NR	NR
Liao et al., 2021	Hyperthyroidism	FAERS	Axitinib	ROR	1.79	1.08	2.98	NR	NR	NR
Liao et al., 2021	Hyperthyroidism	FAERS	Cabozantinib	ROR	2.8	2.04	3.83	NR	NR	NR
Liao et al., 2021	Hyperthyroidism	FAERS	Lenvatinib	ROR	3.28	2.23	4.82	NR	NR	NR
Liao et al., 2021	Hyperthyroidism	FAERS	Pazopanib	ROR	2.94	2.29	3.79	NR	NR	NR
Liao et al., 2021	Hyperthyroidism	FAERS	Ponatinib	ROR	2	1.14	3.54	343	135	596
Liao et al., 2021	Hyperthyroidism	FAERS	Regorafenib	ROR	2.55	1.58	4.11	11	5	39
Liao et al., 2021	Hyperthyroidism	FAERS	Sorafenib	ROR	1.72	1.19	2.48	NR	NR	NR
Liao et al., 2021	Hyperthyroidism	FAERS	Sunitinib	ROR	2.78	2.27	3.42	NR	NR	NR
Liao et al., 2021	Hyperthyroidism	FAERS	Vandetanib	ROR	-			NR	NR	NR
Liao et al., 2021	Hypothyroidism	FAERS	Axitinib	ROR	7.44	6.09	9.09	NR	NR	NR
Liao et al., 2021	Hypothyroidism	FAERS	Cabozantinib	ROR	5.54	4.63	6.63	NR	NR	NR
Liao et al., 2021	Hypothyroidism	FAERS	Lenvatinib	ROR	13.47	11.54	15.72	12	4	32
Liao et al., 2021	Hypothyroidism	FAERS	Pazopanib	ROR	2.71	1.01	7.23	NR	NR	NR
Liao et al., 2021	Hypothyroidism	FAERS	Ponatinib	ROR	2.86	1.96	4.18	429	46	947
Liao et al., 2021	Hypothyroidism	FAERS	Regorafenib	ROR	3.52	2.55	4.87	NR	NR	NR
Liao et al., 2021	Hypothyroidism	FAERS	Sorafenib	ROR	3.09	2.48	3.84	NR	NR	NR
Liao et al., 2021	Hypothyroidism	FAERS	Sunitinib	ROR	11.53	10.6	12.54	NR	NR	NR
Liao et al., 2021	Hypothyroidism	FAERS	Vandetanib	ROR	2.71	1.01	7.23	NR	NR	NR
